# Completing and Balancing Database Excerpted Chemical Reactions with a Hybrid Mechanistic-Machine Learning Approach

**DOI:** 10.1021/acsomega.4c00262

**Published:** 2024-04-10

**Authors:** Chonghuan Zhang, Adarsh Arun, Alexei A. Lapkin

**Affiliations:** † Department of Chemical Engineering and Biotechnology, University of Cambridge, Philippa Fawcett Drive, Cambridge CB3 0AS, U.K.; ‡ Cambridge Centre for Advanced Research and Education in Singapore, CARES Ltd., 1 CREATE Way, CREATE Tower #05-05, Singapore 138602 Singapore; § Chemical Data Intelligence (CDI) Pte., Ltd., 9 Raffles Place #26-01, Republic Plaza, Singapore 048619 Singapore

## Abstract

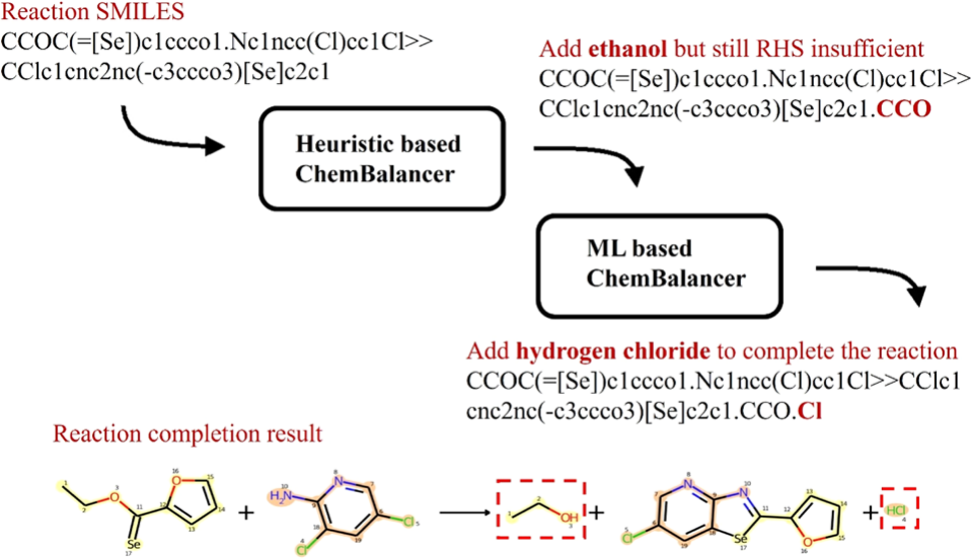

Computer-aided synthesis planning (CASP) development of reaction routes requires an understanding of complete reaction structures. However, most reactions in the current databases are missing reaction coparticipants. Although reaction prediction and atom mapping tools can predict major reaction participants and trace atom rearrangements in reactions, they fail to identify the missing molecules to complete reactions. This is because these approaches are data-driven models trained on the current reaction databases, which comprise incomplete reactions. In this work, a workflow was developed to tackle the reaction completion challenge. This includes a heuristic-based method to identify balanced reactions from reaction databases and complete some imbalanced reactions by adding candidate molecules. A machine learning masked language model (MLM) was trained to learn from simplified molecular input line entry system (SMILES) sentences of these completed reactions. The model predicted missing molecules for the incomplete reactions, a workflow analogous to predicting missing words in sentences. The model is promising for the prediction of small- and middle-sized missing molecules in incomplete reaction records. The workflow combining both the heuristic and machine learning methods completed more than half of the entire reaction space.

## Introduction

**Figure 1:**
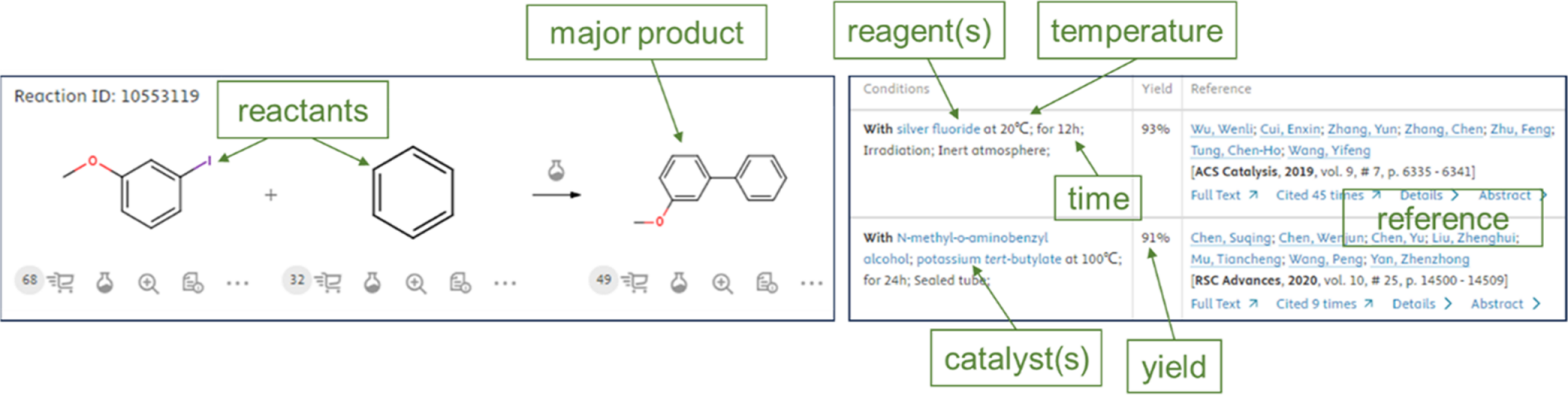
Example reaction record (Reaxys reaction ID: 27812599) summarized from multiple literature sources. The reaction record shows only major reactants and products, while the reaction coparticipants are missing. The reaction participants, which do not contribute to the carbon flow, may be recorded as reagents.

To enable the evaluation of reaction routes with respect to a set of target parameters, such as overall yield, impurities, economy, or greenness, knowledge of the complete reaction record is required. When all reaction participants (reactants, reagents, and products) are known, the reaction completion is simply a problem of mass conservation, i.e., using linear algebra to balance the reaction with stoichiometric coefficients. However, this is not always the case for data records that are currently accessible in Reaxys,^1^ USPTO,^2^ or any other non-manually curated reaction databases. An example of a Reaxys reaction data record is shown in Figure 1. Three main aspects of the reaction data are often missing in records today:^3^ stoichiometric coefficients, reaction coparticipants, and integration of multiple reaction steps into a single reaction entry.

There are historical and habitual reasons for the incompleteness of reaction data today. First, chemists report reactions in journal articles and patents based on the self-defined scope of research, which does not typically have potential tasks of others in mind; side products are either not in the scope of studies or were not detected by the analytical techniques that were used. Second, although text-mining techniques could process chemical information from the literature, including properties and structures of molecules and reaction conditions, sometimes it is hard to identify reaction participants as they may appear in different sections of a publication. This aspect is being addressed by creating clear templates for presenting and storing reaction data, such as the Open Reaction Database project.^4^ For data to be useful for machine learning tasks and for automated tasks of process development, it is necessary for existing data to be recalibrated to include the missing reaction participants and reduce the noise in the data sets.

In the literature, several methods for reaction structure completion were published. Grzybowski et al. manually curated around 100,000 generalized reaction rules with complete understanding of reaction participants and stoichiometry.^5^ Their templates are now linked with commercial software SYNTHIA and can guide retrosynthesis and analyze carbon efficiency based on mass conservation.^6^ However, human development of reaction rules is far from the aspiration of exploring a very large chemical space of feasible molecules and reactions. On the other hand, the automatically extracted templates^7^ are also not reliable for reaction completion since most of these templates were generalized from open-source USPTO reaction data set^2^ in which the reactions were not complete. A “golden data set” with complete reactions could be an ultimate solution. However, such a large-scale reaction data set does not exist today.

Atom mapping, which relies on the rearrangement of atoms in chemical transformations, is promising to tackle this problem. Atom mapping describes the exact transformation and reveals the missing species on the product side. Jaworski et al. utilized graph-theoretical considerations and chose 20 chemical rules/heuristics to the correct mapping of reactions.^8^ This method attempts to complete stoichiometry first by adding small molecules such as acetaldehyde, ammonia, and others to balance the reactions and, second, by fitting reactions into popular reaction templates and adding the missing parts. Only if such attempts fail is atom mapping employed.

The atom mapping tool RXNMapper developed by Schwaller et al. utilized NLP to infer reaction structures.^9^ A neural network (transformer) was trained on a set of mapped reactions and proved to be capable of completing the mapping tasks quicker and with confidence scores.^9^ Nugmanov et al.^10^ developed a rule-based reaction balancing method, and this has been integrated into reaction informatics software, namely, CGRtools.^10^ However, this could only add small molecules, such as water, and the author claimed that the balancing was imperfect. Gillet et al.^11^ used a knowledge base of chemical reactions represented as reaction vectors to balance unbalanced reactions. By selecting combinations of reaction vectors that collectively satisfy stoichiometry constraints, the algorithm optimized the reaction balance. However, this method struggled with reactions involving uncommon chemical reactions not adequately represented in the knowledge base. Thus, until now, inferring complete reaction structure from incomplete reaction data sets remains a challenge.

In this work, we focused on the existing reaction data records in the databases and aimed to add substances to the left- and right-hand sides of the reaction records to complete the chemical equations with correct stoichiometry. A workflow was proposed as follows. Given a reaction record, either from reaction Simplified Molecular Input Line Entry System (SMILES),^12^ which is a textual representation of chemical structures used in computational chemistry and cheminformatics, or other reaction representations, a proposed heuristic tool, ChemBalancer, first checks if the reaction is a complete reaction or incomplete from the left-hand side (LHS) or the right-hand side (RHS) of the reaction equation. The ChemBalancer intends to add one (or sometimes more than one) specie into a side (or sometimes two sides) of reaction accordingly, with the aid of reaction atom mapping tool RXNMapper.^9^ If the ChemBalancer fails to complete the reaction record, the reaction SMILES string is considered as a language of chemistry and is passed into a fine-tuned masked language model (MLM), a BERT transformer,^13^ originally designed to detect missing words in a sentence based on its context, to infer the most possible missing molecule in the reaction. Here, the MLM model, ChemMLM, was trained from a set of completed reactions detected by ChemBalancer to predict the missing molecules in a reaction. This is followed by using ChemBalancer to further check if the updated reaction is completed. Every step of this pipeline intends to only add one most possible molecule to the incomplete reaction, and the specific sequential design of this pipeline was to learn only from available complete reactions and maximize the likelihood to propose correct molecules for incomplete reactions toward reaction structure completion.

## Methods

### The Heuristic Step: ChemBalancer

**Figure 2:**
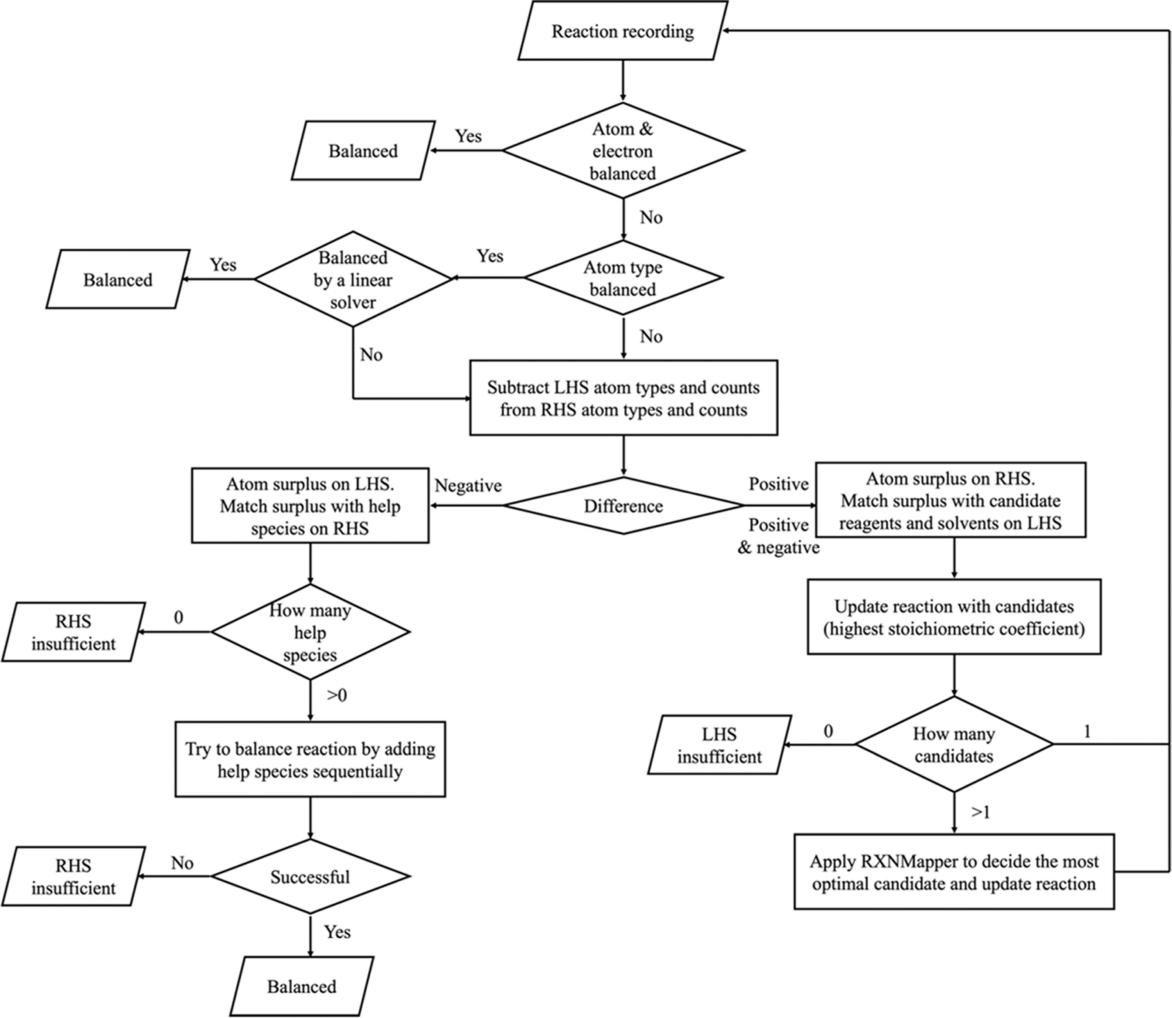
Scheme of the ChemBalancer workflow to detect if reaction records are originally complete or could be completed with help species added to the RHS or candidate species added to the LHS or the reactions are eventually incomplete with LHS or RHS species insufficient.

ChemBalancer was partially adapted from Arun et al.’s balancing algorithm,^14^ which was originally proposed as a step to identify chemical impurities produced in reactions. The workflow of ChemBalancer is shown and summarized in Figure 2.

A reaction is defined as complete if atoms of each element and electron charges are balanced between the LHS and RHS of a reaction equation. Hence, for a given reaction record, the first step for the ChemBalancer is to determine the atom and the electron balances. By default, ChemBalancer ignores the counts of hydrogen atoms since from the perspective of solving linear algebra of reaction balancing, a valid reaction with mass conservation and all other atoms and electron charges balanced between LHS and RHS cannot give a degree of freedom for hydrogen imbalance. Hydrogen atoms should have already been balanced between LHS and RHS. If a reaction imbalance includes atom and electron counts but the types (chemical elements) are identical, this reaction can be balanced with stoichiometry. A linear solver in ChemPy API^15^ was used to determine stoichiometric coefficients. In some rare cases, large values of stoichiometric coefficients are added to the reaction participants. Since the chance of having large numbers of molecules react together to trigger a reaction is low, an arbitrary value of six was set as an upper limit for stoichiometric coefficients. Here, we must differentiate the small molecule synthesis from polymerization reactions, which could be represented by chemical equations with very large stoichiometric coefficients.

If stoichiometry alone cannot solve the reaction balance, ChemBalancer attempts to add possible missing molecules to complete the reaction of interest. To do this, ChemBalancer subtracts the counts of each atom and electron at the LHS from the counts at the RHS.

If this results in an atom surplus on the LHS, one compatible small help species is added to the RHS. A library of manually curated help species was tailored to include 32 molecules and ions ranging from water to chlorobenzene. These molecules are the most frequently appearing side products in organic synthesis. The full list of help compounds is shown in Table S1 in the Supporting Information (SI). These help species are added sequentially to the RHS of a reaction mixture and used to balance the reaction. The reaction is defined as complete with help species if it is balanced with the addition of these species to the RHS, as shown in the “successful” block of Figure 2. This means the reaction is either “successful” with “atom and electron balanced” or “balanced by a linear solver” condition. Otherwise, it is classified as a “RHS species insufficient” reaction.

If this step presents an atom surplus on the RHS, this means that at least one reactant is missing from the reaction. The most likely reactants that may complete reactions are listed as reagents or solvents in the reaction record. For example, in Reaxys, reagents, i.e., reactants that do not contribute to the carbon flow of the reaction, and solvents are listed as two attributes in the reaction record, as shown in Figure 1. In USPTO, reactions are given with reaction SMILES strings in the format of “A.B > C.D > E.F”, in which “C” and “D” are reagents, while solvents are not provided. ChemBalancer picks a molecule from the candidate reagents and solvents to add to the LHS of the reaction. If the reaction has multiple candidate reagents and solvents, the atom mapping tool RXNMapper^9^ is implemented to map the atom rearrangement from the reactants to the products for each candidate reaction. RXNMapper can trace the reactant’s origin of each atom of the product. Given a reaction with RHS atom surplus, this helps identify the percentage of product atoms that can be traced with their origins at the LHS, and this is quantified by the atom mapping confidence score.^9^ The confidence scores for the candidate reactions are ranked to select the most optimal candidate molecule to update the LHS of the reaction. ChemBalancer treats the updated reaction as a new record and tries to balance the updated reaction again with the same workflow. This corresponds to the arrow from the bottom-right box to the top box in Figure 2.

Each loop of ChemBalancer aims to add one possible missing molecule to the reaction mixture to complete it. However, in the case when a candidate reagent or solvent is added to the LHS, the reaction reaches a new imbalance status. If it is passed into the workflow in a new balancing loop, then this may potentially result in adding more than one molecule to both sides of the reaction. The reaction is eventually defined as “LHS insufficient” reaction when there are no available reagents or solvents to be added to the LHS to balance it.

If a reaction has an atom surplus on the LHS for some atom types and an atom surplus on the RHS for others, this means the reaction is missing both, at least one product and one reactant. With given candidate reagents and solvents, the reaction completion starting from adding species into the LHS becomes more certain and has a higher priority than the RHS. Therefore, this case follows the same procedure as the reaction with atom surplus on the RHS. However, since atom surplus exists on both sides of the reaction, the possibility of adding only candidate reagents and solvents on the LHS to complete the reactions becomes very low. Therefore, help species are also allowed to be added to the LHS as well but with a lower priority than the candidate reagents and solvents since they are prone to create false positive complete reactions.

### The Machine Learning Step: ChemMLM

Those reactions that were failed to be balanced by ChemBalancer were marked as incomplete reactions and passed to ChemMLM to infer the next most possible molecules in the LHS or the RHS of the reaction record based on conclusions with regard to LHS or RHS insufficient species reached by ChemBalancer.

Several assumptions were made in developing ChemMLM:

1. All results passed from ChemBalancer were assumed to be correct. While the originally balanced reactions verified by ChemBalancer are usually correct, there remain false positives in the reactions completed by ChemBalancer itself. Also, since ChemMLM was trained on a set of completed reactions detected by ChemBalancer, all reactions in this set were assumed correctly completed by ChemBalancer, and they would result in a propagated error in training and reduce ChemMLM accuracy.
2. ChemMLM assumes only one molecule is missing in incomplete reactions. Adding more than one molecule would result in the combinatorial increase of the decision space, which is not guaranteed to lead to ground truth molecules but becomes computationally expensive. In this case, ChemMLM was trained to predict the most possible next molecule to be added to the reaction toward reaction completion. However, we noticed that sometimes ChemMLM predicts more than one molecule in the SMILES format, using representation “A.B”. This is discussed in the following sections.

#### The ChemMLM Model Structure

MLM is an application of BERT transformer.^13^ Using BERT to learn from many linguistic patterns of complete sentences, the MLM predicts missing words in a sentence inferred from its context. For example, to fill the gap “In autumn, the ___ falls from the trees”, MLM restricts search to the most possible item falling from trees in autumn, which would be the word “leaves”. To train an MLM, a BERT transformer is fed with the same input sequence as the output, and the model optimizes weights within its encoder layers in order to process this. To predict a missing word, tokens are randomly masked within the input sequence by replacing the missing word token with a special mask token symbol “⟨mask⟩”. Given the context of a sentence, the MLM trained on previous semantic examples would infer the possible missing word.^16^

**Figure 3:**
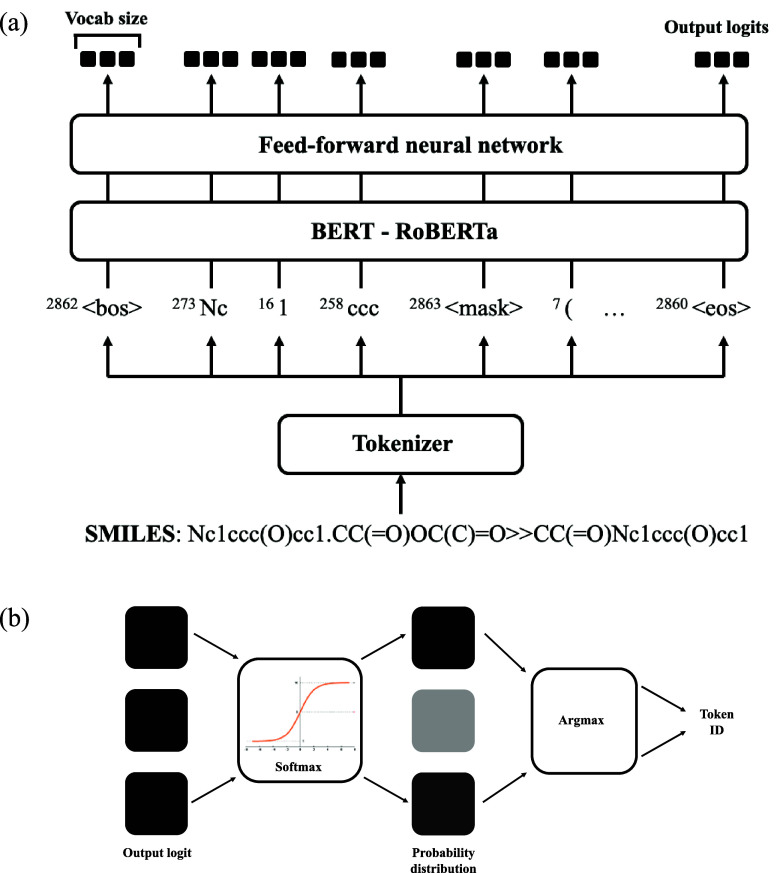
Model structure of ChemMLM: (a) transformation of reaction SMILES strings into output logits by passing the tokenizer and the RoBERTa model, using an example of reaction SMILES of paracetamol synthesis and acetylation of 4-aminophenol with acetic anhydride; and (b) transformation of output logits into token IDs by passing softmax and argmax functions.

Figure 3 shows a proposed model structure for ChemMLM adapted from an MLM model.^16^ In Figure 3a, reaction SMILES strings are processed into ChemMLM. A reaction SMILES is tokenized into multiple tokens and replaced by index IDs that have a one-to-one correspondence with the tokens. The reaction SMILES tokenization method is discussed in the ChemMLM Tokenizer section of this paper. A mask function dynamically masks a section of reaction SMILES, where the mask method is discussed in the Mask Method section of this paper.

The input ID tensor is processed by a BERT transformer model, where the RoBERTa^17^ variant of BERT is adopted. The theories and model architecture of RoBERTa are discussed in the RoBERTa Model section of this paper. RoBERTa outputs a set of vectors with a length of 768, and each vector is transformed from a token by RoBERTa. The vectors are passed into a feed-forward neural network, which outputs the output logits. The output logit is the logit transformation applied to its original output, shown in eq 1. The output vector of each token remains the size equal to the vocabulary size, i.e., the total number of tokens, indicating the projection of each token into each output logit
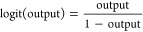
1The step to convert each set of output logits into an output token is shown in Figure 3b. From the output logits, a softmax transformation is applied to acquire probability distribution of a list of possible predicted tokens. This is followed by applying an argmax function to select the most possible index ID for the token. Concatenating the strings of tokens produces a predicted reaction SMILES, which reveals the masked tokens represented in the mask symbol “⟨mask⟩”.

The input tokens are compared with the predicted tokens through the Kullback–Leibler (KL) divergence loss function, shown in eq 2, which pairwise measures the probability distribution of the true output encoding *y*_true_ and computed one *y*_pred_. The loss is backpropagated to the encoder layers of RoBERTa, the feed-forward neural network layers, and the softmax function to update the weights.
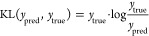
2

#### The ChemMLM Tokenizer

Ideally, in an MLM model, each word is converted into a single token, and prediction of a missing word is the prediction of one token. However, this could not be the case for ChemMLM. With 160 million molecules present in the database, the conversion of a single molecule into a token would result in a vocabulary size of 160 million, which was impossible to process in ChemMLM.

A well-known regularized reaction SMILES tokenization method has been proposed by Schwaller et al.^18^ to deliver the promising reaction prediction tool—Molecular Transformer. In this method, all atoms and regularly used expressions in reaction SMILES are separated into tokens, such as “C”, “Br”, “@”, and “)”. Although this regularized tokenization was implemented in multiple related works, it was not used here. This approach discretizes a molecule into multiple tokens, which makes it harder to learn from the semantic context of a molecule’s SMILES string.

A byte-pair encoding (BPE) tokenization method^19,20^ was used in this work. The BPE tokenization counts the highest frequent consecutive string expressions of reaction SMILES to replace with tokens. For example, in Figure 3a, “Nc” and “ccc” are both highly frequent expressions in reaction SMILES, and therefore, they were formed as single tokens. This tokenization method disconnected molecules at various positions, which allowed the model to learn different molecular disconnection strategies, and this also steered the model to infer various types of missing molecules.

Highly frequent expressions were usually present within the molecule representations of the reaction SMILES, i.e., the splitting molecule symbol “.” and the splitting reactant–product symbol “≫” do not exist in the tokens. However, in several cases, tokens were formed across the molecule representations since the BPE tokenization method recognizes SMILES strings of “part of a molecule’s SMILES +. + part of another molecule’s SMILES” as highly frequent expressions, such as “+].[” and “.[”.

Four special symbols were added into the token vocabulary, which are “⟨bos⟩”, “⟨eos⟩”, “⟨mask⟩”, and “⟨pad⟩”, meaning, respectively, the start token of a reaction SMILES string, the end token of a reaction SMILES string, the mask token, and placeholder of void tokens. Using all Reaxys and USPTO reactions as the semantic input, the BPE method tokenized the reaction SMILES expression into 2,863 tokens including the four special tokens.

#### RoBERTa Model

RoBERTa model^17^ builds on the BERT transformer^13^ and modifies key hyperparameters of the BERT transformer. The choice of RoBERTa over the original BERT here was because of its use of the BPE tokenization approach, as discussed in the ChemMLM Tokenizer section, and dynamic mask method, as discussed in the Mask Method section. The other architecture of RoBERTa is close to that of BERT.

In Figure 3, the “BERT-RoBERTa” unit includes the input embedding unit, positional encoding unit, multihead attention units, and normalization layer, followed by layers of feed-forward neural network. These units enable the ChemMLM to encode the semantic and syntactic information on the reaction SMILES in the embeddings.

#### ChemMLM Model Training and Prediction

##### Data Source

Since a “golden data set” with a large range of complete reactions did not exist at the start of this work, all accessible complete reactions were valuable data sources, despite doubts about data quality of these complete reactions. The objective was to explore the limited complete reaction space and to broaden the boundary of the space to some originally incomplete reactions while retaining errors and noise present in the space. Therefore, the complete reactions, i.e., the originally complete reactions detected by ChemBalancer and the originally incomplete reactions balanced by ChemBalancer, were used to train the ChemMLM model. Reaxys and USPTO reaction data sets were two accessible reaction data sources. Although the data sizes of the two data sets are in different magnitudes—approximately 21 million versus one million, they both cover broad ranges of reaction types,^21^ which are both key data sources to learn from.

To compare the model predictability from different data sources, ChemMLM models were trained from the complete reactions from (i) USPTO data set and (ii) Reaxys plus USPTO data set (this is referred to as the combined data set in the rest of this paper). Since the magnitude of USPTO is not comparable with Reaxys, it was added to Reaxys as a whole data source to train the second ChemMLM model.

The data was randomly split into training, validation, and test data following the ratio of 9:0.5:0.5 to train, evaluate the potential model structure, and understand the model predictability, respectively. We did not split this manually based on the reaction classes/SMILES lengths since the reaction data set is large enough to cover different types/SMILES lengths of reactions into different data sets in random split. Only a small portion of reactions were used for model validation and test since the total number of reactions is large.

##### Mask Method

A dynamic masking method randomly masked approximately 15% of the tokens in the input ID tensor. The number of masked tokens was rounded based on the number of tokens in the reaction SMILES. To avoid using the same masks for every epoch of training, the method randomly masked the reaction SMILES iteratively in every epoch to increase the exposure of every token as a mask.

##### Model Implementation

The ChemMLM models were implemented with a Python API, namely, Face,^22^ which is a platform to implement multiple NLP transformer variants. The training calculations of the ChemMLM models were powered by Google Colab’s GPU cloud service.^23^

The model structure details configured for the RoBERTa model are shown below. The dimensionality of the encoder layers is 768. The maximum token length that the model can be used with is 512, which covers all reaction SMILES token lengths (up to 333). In the encoder, the number of attention heads for each attention layer is 12, and the number of hidden layers is 6. The activation function used in the model is Gaussian Error Linear Units (GELU) function.^24^ The dropout probability for all fully connected layers in the embeddings and encoder is 0.1, and the dropout ratio for the attention probabilities is 0.1. The epsilon used by the normalization layers is 10^–12^. The configuration of such a model creates 83,504,416 parameters.

After fine-tuning of ChemMLM training arguments based on the validation data results, the model arguments for the final ChemMLM models trained from USPTO data and combined data are shown in Table S2 in the SI, and “adam” optimizer was used to optimize the model parameters.

##### Model Test Method

The trained ChemMLM models were assessed on the reaction test data set. The model used KL divergence loss, determined in eq 2 between predicted tokens and the true masked tokens, to backpropagate the model parameters and validate the models. However, this assessment could only conclude on the model predictability of a masked token, which is usually part of a molecule. The purpose of the model testing was to assess whether the model could infer an entire missing molecule, rather than a masked token. Moreover, since the model predicts the missing molecule for the incomplete reactions following the LHS and RHS species insufficient scenarios, as discussed in the Missing Molecule Prediction section, the ChemMLM models were tested, with respect to their LHS and RHS predictability.

With respect to model predictability testing at the RHS, in each complete reaction SMILES string in the test data set, each product was hidden once alternatively with the missing molecule symbol “@@@”. For example, a reaction with the format of “A.B.C ≫ D.E” would be duplicated into two reaction SMILESs, “A.B.C ≫ @@@.E” and “A.B.C ≫ D.@@@”. With respect to the LHS testing, in each reaction, each reactant was hidden once alternatively. The example reaction would be duplicated into three reaction SMILESs, “@@@.B.C ≫ D.E”, “A.@@@.C ≫ D.E”, and “A.B.@@@ ≫ D.E”. In this way, the number of reactions in the test data set was also augmented, and the LHS ChemMLM and the RHS ChemMLM were used to predict the hidden molecules on each side, respectively.

Each “@@@” symbol was converted to the multiple of the mask symbol “⟨mask⟩” based on the number of tokens included in the hidden molecule. In this way, instead of dynamically randomizing 15% of the mask, the masks in the test data set were tailored to mask an entire molecule sequentially at the designated side of the reaction. The ChemMLM model predicted the masked tokens and concatenated the tokens to predict SMILES of the hidden molecule.

Any deviation in tokens between the predicted molecule and the ground truth would make the prediction of the molecule semantically meaningless. Therefore, instead of comparing the KL divergence loss, as used in model training and validation, SMILES of the predicted molecules and the ground truth molecules are compared directly to determine the correction rate of identical SMILES strings among the test data set. For each ChemMLM model, two values were computed, i.e., the LHS and the RHS correction rates in the test data set.

The hidden molecule mask method was also initially considered as a regularized mask method to mask molecules during training instead of the dynamic mask approach. However, such a mask method hides a molecule at a centralized segment (i.e., one single token) of the reaction SMILES. By no means could the mask method learn bond breaks and recombination from the reaction SMILES, and it was hard to pick the semantic context among the molecules. Therefore, a dynamic mask approach was implemented.

##### Missing Molecule Prediction

**Figure 4:**
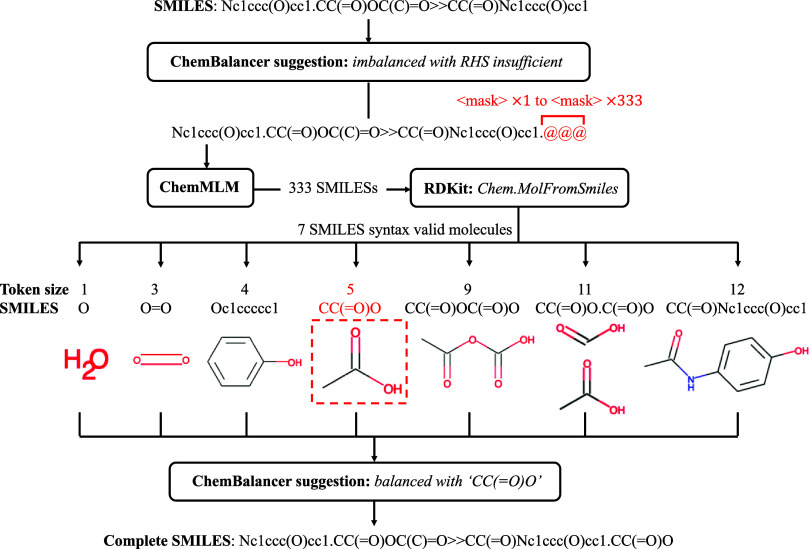
Illustration of the workflow to predict the missing molecule for an incomplete reaction, illustrated with an example of an RHS species insufficient reaction, paracetamol synthesis. The reaction is completed with an acetic acid molecule added to RHS of the reaction.

Based on the suggestion from ChemBalancer, a reaction is imbalanced with either LHS or RHS species insufficient. Assuming only one molecule is missing in the reaction SMILES, an extra missing molecule symbol “@@@” of a missing molecule is added to the corresponding side of the reaction SMILES. For the paracetamol synthesis discussed above, ChemBalancer suggested RHS species insufficient for the reaction, and therefore, “@@@” symbol was added to the end of the reaction SMILES RHS, split by splitting the molecule symbol “.” from the original reaction SMILES, shown as “Nc1ccc(O)cc1.CC(=O)OC(C)=O ≫ CC(=O)Nc1ccc(O)cc1.@@@”. This example is shown in Figure 4.

The prediction of a known hidden molecule from a complete reaction is different from the prediction of a missing molecule from an incomplete reaction. While the number of tokens of the hidden molecule from the complete reaction is given, that of the missing molecule from an incomplete reaction remains unknown. A molecule in USPTO and Reaxys reactions can have token lengths ranging from one to 333. The 333 scenarios are all enumerated by converting the missing molecule symbol “@@@” in reaction SMILES into different numbers of mask symbols “⟨mask⟩”. For example, for the scenario of the missing molecule with two tokens, the paracetamol synthesis reaction SMILES is converted to “Nc1ccc(O)cc1.CC(=O)OC(C)=O ≫ CC(=O)Nc1ccc(O)cc1.⟨mask⟩⟨mask⟩”.

ChemMLM was used to predict the masked tokens and concatenate them into a molecule. It is noticed that in some cases, more than one molecule was concatenated from the masked tokens since the model can predict crossing-molecule tokens, which split the concatenated reaction SMILES tokens into multiple molecules.

From the 333 prediction enumerations, only one token length could potentially give the ground truth for the missing molecule, and the other 332 enumerations are semantically meaningless predictions, and even sometimes, the SMILES syntax of the predictions is incorrect. To determine the meaningful result, the RDKit API^25^ was used to convert the 333 candidate molecular SMILES strings into molecule objects, and the syntax meaningless molecules would throw errors and be removed from the candidate molecules. For example, for the paracetamol synthesis reaction, only 7 out of the 333 enumeration produce SMILES syntax valid molecules, which have the token lengths of 1, 3, 4, 5, 9, 11, and 12. Among these scenarios, the 11 tokens concatenate into two molecules, with “.” presented in the SMILES string, as shown in Figure 4.

**Figure 5:**
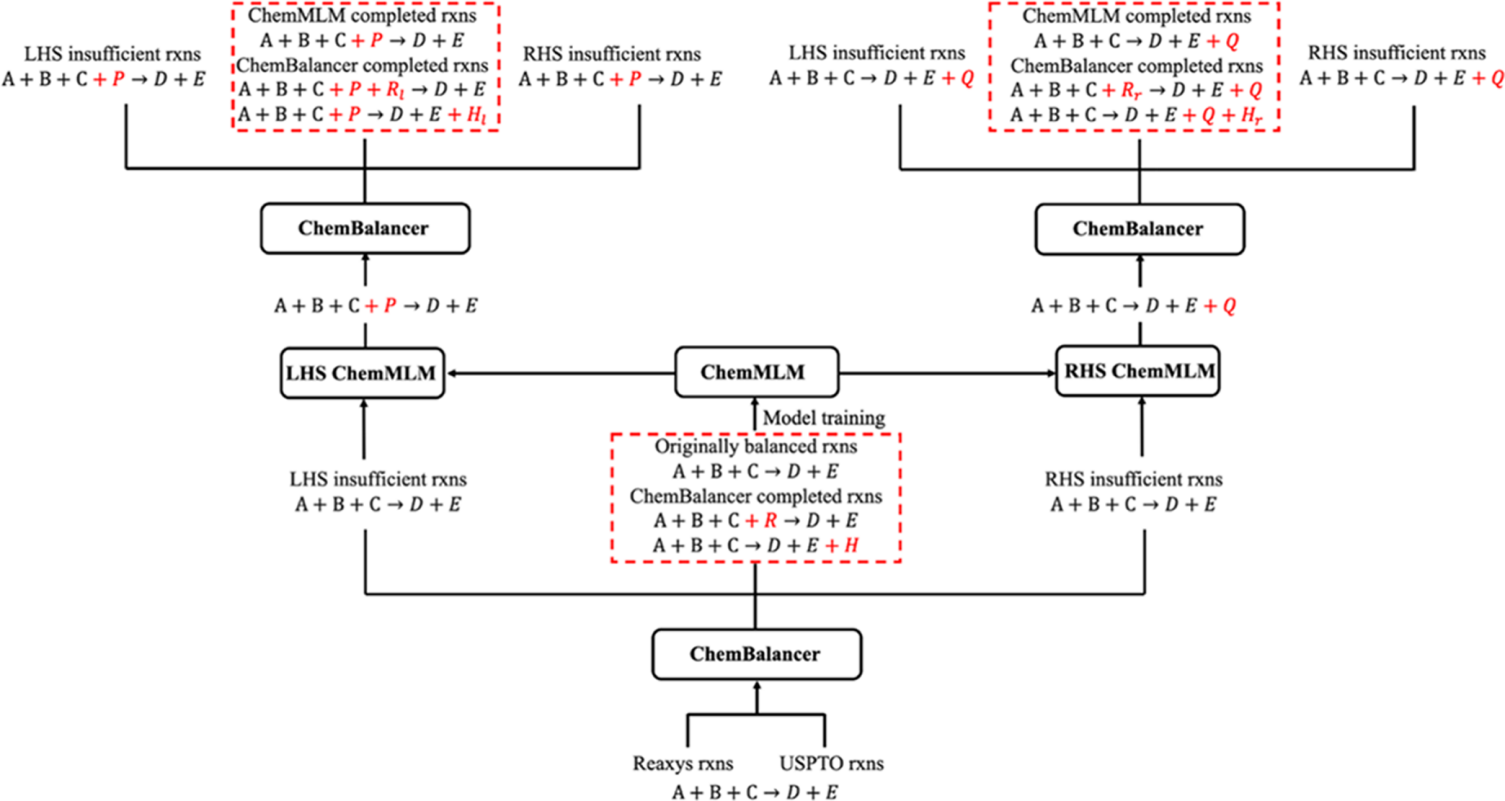
Illustration of the entire workflow of the reaction completion algorithm, including ChemBalancer and ChemMLM subunits, exemplified by a reaction scheme “A + B + C → D + E”. All complete reactions are shown in red dashed boxes.

These syntax valid molecule SMILES replace the “@@@” in the reaction SMILES and are passed to ChemBalancer to determine if these candidate molecules include a chemically meaningful molecule that could either complete the reaction itself or could balance the reaction with extra molecules proposed by ChemBalancer. If both scenarios fail, this means the reaction could not be completed with the current workflow, as shown in Figure 5.

### Reaction Completion Workflow

The entire workflow of the reaction completion algorithm includes two sequential subunits, ChemBalancer and ChemMLM. The workflow is summarized in Figure 5. In the ChemBalancer subunit, the USPTO and Reaxys reactions were passed to ChemBalancer to determine if each reaction, exemplified by a reaction scheme “A + B + C → D + E”, is originally complete or could be balanced with ChemBalancer by adding a candidate reagent “R” or a help compound “H” into the reaction or sometimes combinations of “R” and “H”. The complete reactions shown in the bottom red dashed box of Figure 5 were used to train the ChemMLM model.

In the ChemMLM subunit, for the ChemBalancer-determined LHS insufficient reactions, the trained ChemMLM proposes multiple solutions for an extra reactant “P”. ChemBalancer then detects which proposed reaction “A + B + C + P → D + E” is balanced or could be balanced by adding a reagent “R to the LHS or a help compound H_1_ to the RHS or sometimes combinations of R_1_ and H_1_. Similarly, for the RHS insufficient reactions, ChemMLM proposes multiple solutions for an extra product Q. ChemBalancer then detects which proposed reaction A + B + C → D + E + Q is balanced or could be balanced by adding a reagent R_r_ or a help compound H_r_ or sometimes combinations of R_r_ and H_r_. When multiple completion solutions are proposed for a given reaction, the atom mapping tool RXNMapper selects the most optimal solution based on the highest-ranked confidence score on atom mapping.

All of the complete reactions are shown inside the red dashed boxes in Figure 5. These reactions could potentially be used to train another round of the ChemMLM model for further broadening the complete reaction space. However, this was not conducted since the workflow carries erroneous assumptions. These errors would be propagated into another round of training if these data were used to retrain the ChemMLM model and further reduce the predictability.

## Results and Discussion

### The ChemBalancer Completion Results

ChemBalancer attempted to complete the reaction structures from the available resources (the candidate reagents and solvents and the help species) by a heuristic approach. All reaction records from Reaxys and USPTO databases were passed to ChemBalancer, with their completion results shown in Table 1. In USPTO and Reaxys, 17.6 and 44.1% of reaction records were complete with ChemBalancer, respectively. These complete reactions include the originally complete reactions, the reaction completed by adding candidate reagents to the LHS (or reagents to the LHS and help species to the RHS), and the reaction completed by adding help species to the RHS. Table 1 also shows the ratio of reactions failed to be completed by ChemBalancer, with species insufficient at the LHS or RHS of the reactions. The reactions that have both atom surplus in the LHS and RHS of the reactions were classified into the LHS species insufficient reactions since the priority to add species to the LHS was higher than RHS. The majority of the incomplete reactions are RHS species insufficient.

**Table 1:** Statistics for the Reaction Completion Results by ChemBalancer for the Reaction Records in the USPTO and Reaxys Databases

reaction database	USPTO	Reaxys
complete reaction	17.6%	44.1%
--originally complete reactions	3.3%	7.2%
--completed with reagents	0.5%	6.2%
--completed with help species	13.8%	30.7%
LHS species insufficient	2.1%	13.4%
RHS species insufficient	80.3%	42.5%
total complete reactions	171,637	7,043,030

It is noticed that ChemBalancer has better performance on Reaxys reactions over USPTO. A very high percentage of reactions in the USPTO remains incomplete due to their RHS species insufficiency. This means that most reactions could not identify their side products from the library of help species. A larger set of help species was assembled to have more diverse solutions to complete the reactions. This list included 3526 frequently appearing small species. Additionally, from a set of LHS atom surplus reactions, enlarging the library of help species from 32 to 3526 frequently appearing molecules as reaction side products was also attempted. However, this could only improve the reaction completion rate by 0.3% but significantly increased the computational time for the enumeration. This suggests that the current 32 help species are sufficient to comprise the help species library. This also indicates a case-specific heuristic method or predictive machine learning is preferred to predict the exact side products for the RHS insufficient reactions. It is also noticed that the ratio of reactions with RHS species insufficiency is much higher in the USPTO reactions. This might be because the USPTO reaction extraction algorithm^2^ incorrectly added reagents into the reaction SMILES as reactants. Using the atom mapping tool RXNMapper, it is noticed that the reactants from a great number of USPTO reactions have no atoms mapped into the reaction products. With no contribution to the carbon flow, these reactant molecules were supposed to be added to the reagent categories. Adding these into reactants would cause LHS atom surplus and therefore result in RHS species insufficiency. The USPTO complete reaction ratio would be higher if these false-labeled reactants could be removed. It is also noticed that RXNMapper sometimes struggles with symmetric reactions, which give incorrect atom mapping results. Some potential ways forward could involve detecting these types of reactions via heuristics (e.g., check product and reactant similarity and inspect reactions past a certain threshold). Devising some general rules for balancing these types of reactions would be the next step (although it depends on how many examples there are). Also, inspecting how many atoms are unmapped or if there are sequences of mapped atoms in the products where reactant ownership rapidly changes could be other ways to detect flawed mappings in these cases.

**Figure 6:**
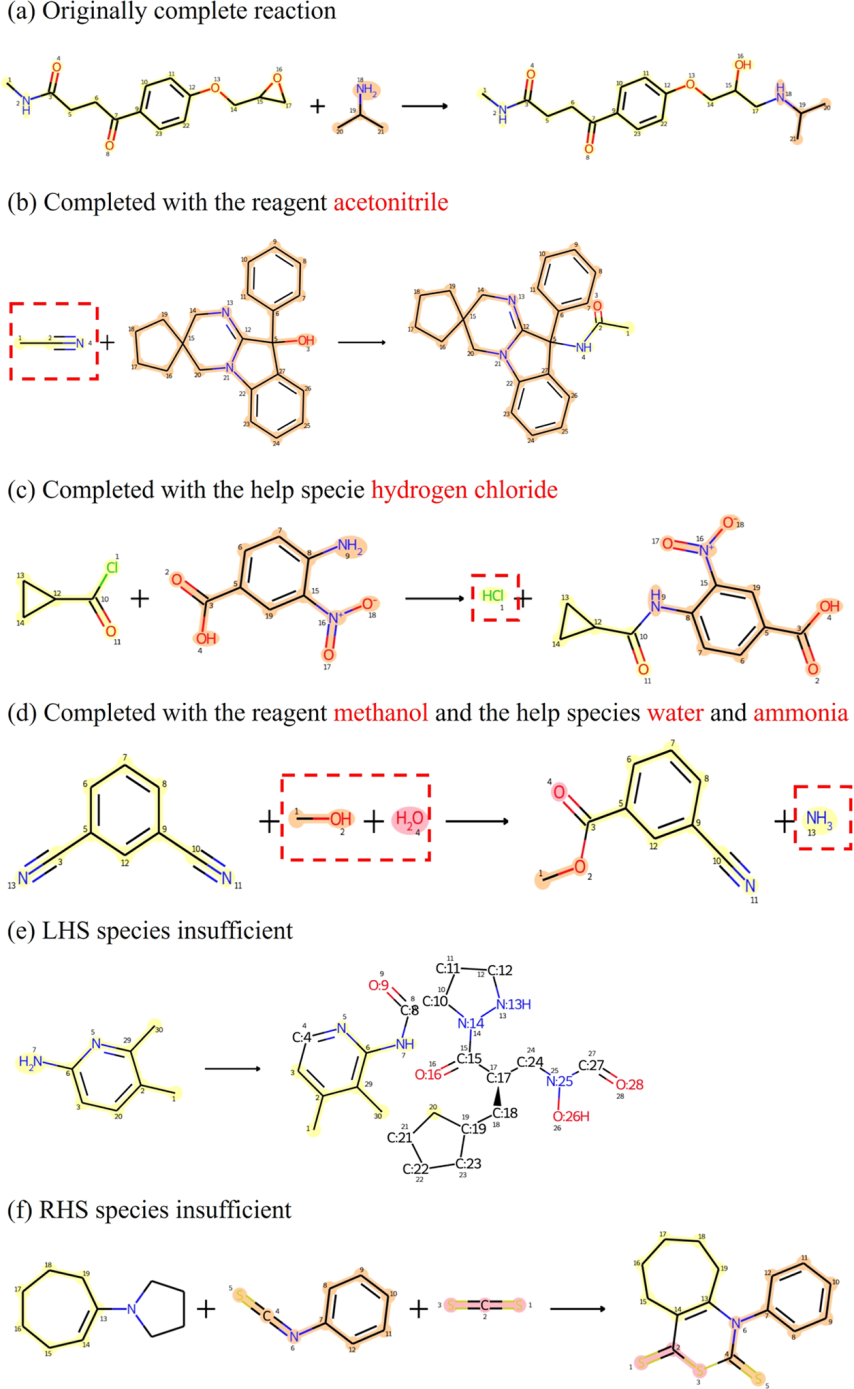
Examples of ChemBalancer reaction completion results: (a–d) complete reactions, (e) an LHS species insufficient reaction, and (f) an RHS insufficient reactions. The molecules added by ChemBalancer are shown in the red dashed boxes. All reactions are atom-mapped by the RXNMapper and labeled with atom mapping indices.

ChemBalancer eventually obtains 171,637 and 7,043,030 complete reactions, respectively, in USPTO and Reaxys by removing the redundant reactions and reactions with molecules not sanitizable by RDKit. These reactions cover large reaction spaces from which ChemMLM can learn from. Reaction examples for each completion and incompletion scenarios are shown in Figure 6. The ChemBalancer-added reagents and help species, shown in Figure 6b–d, were evaluated from their atom rearrangement by the atom mapping tool RXNMapper^9^ and also manually by human experts from their reaction mechanisms to be correct predictions for these example reactions. The yellow and pink colors in the figure trace the atom flow of substrates into products. After atom mapping between the substrates and the products, the atoms mapped in the products traced from substrate 1 are highlighted in yellow color, and the atoms traced from substrate 2 are highlighted in pink color.

The reaction given in Figure 6d shows an example of a reaction completion by adding a reagent methanol and a help species water at the LHS and adding a help species ammonia at the RHS. The original reaction record, i.e., the reaction participants outside the red dashed boxes, has an atom surplus of nitrogen at the LHS and an atom surplus of carbon and oxygen at the RHS. ChemBalancer prioritized to first solve the RHS atom surplus by adding one of the candidate reagents and solvents to the LHS. RXNMapper suggested that among the candidates, adding methanol would give the highest mapping confidence score. Afterward, by adding help species of water at the LHS and ammonia at the RHS, the updated reaction was balanced. Help species were only allowed to be added to the LHS when the reaction has atom surplus at both sides. This reaction follows the mechanism of the Pinner reaction, i.e., under an acidic environment, an alcohol esterificates a nitrile with water to form an imino ester.^26^

**Figure 7:**
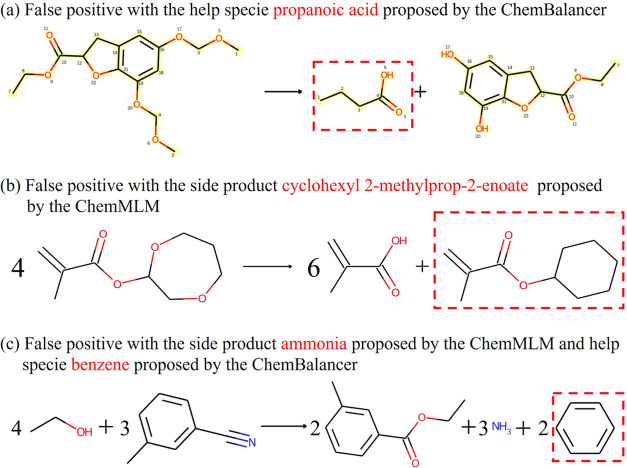
Examples of false positive complete reactions: (a) a false positive ChemBalancer completed reaction, (b) a false positive ChemMLM completed reaction, and (c) a false positive reaction with both ChemBalancer and ChemMLM added molecules. The added molecules are shown in red dashed boxes. Atom mapping cannot be identified for the reactions in parts (b, c).

ChemBalancer balanced a large number of incomplete reactions by adding help species to the RHS of the reactions. Although these help species were the most frequent species present as the side products of the reactions, by adding these help species as side products, these reactions were completed only in terms of material balance at the two sides of the reactions. However, the reaction mechanisms were not evaluated, and therefore, some help species completed reactions remain false positive. In contrast, the reagent- or solvent-balanced reactions were rarely incorrect since these candidate reagents and solvents are clues to complete the reactions given as reaction attributes. Examples of false positive reactions completed by ChemBalancer are shown in Figure 7a. In this reaction, it is clear that the carbon–oxygen bonds (specifically with atom indices, the “C[:3]-O[:17]” and “C[:4]-O[:30]” bonds) of the reactant need to be disconnected by a reagent. However, ChemBalancer only proposes a false positive product, propanoic acid, to the reaction.

There were some safeguards to false positive outcomes implemented in the heuristic step of ChemBalancer. One such was imposing a stoichiometric coefficient limit of 6, as reactions that have high coefficients (other than hydrogenation of nitrile groups/triple bonds) are usually missing species or are incorrectly balanced with existing species. Despite this, false positive outcomes can stem from

(1) Incorrect assignment of possible RHS species that materially balance (with respect to atoms) with low coefficients but do not make sense in the reaction context.
(2) Incorrect selection of LHS reagents, solvents, etc., that participate in the reaction and which materially balance with low coefficients but do not make sense in the reaction context.

We could potentially add a calibration step looking at the mapping score; however, in a few false positive outcomes, the confidence is still high as all atoms are mapped with low stoichiometric coefficients. Additionally, other correctly balanced and mapped reactions have low confidence scores too, so relying on the mapping score as a filter is not a robust, long-term solution and would lead to a sparse data set. One way to avoid the assignment of incorrect RHS species (1) is to take unmapped fragments on the LHS and place them on the RHS as a more intelligent guess. However, reason (2) is still challenging to address, as we still require RXNMapper to make the choice, which itself has been initially trained on imbalanced data.

Looking at broader reaction types and abstracting rules or heuristics for side products that make sense may be a way forward. (In so doing, we would at least avoid manually inspecting each and every outcome but instead reaction types.) Figure S1 in the SI shows a chart with the top 10 most common reaction classes in the USPTO data set we used based on the NameRXN tool developed by NextMove software. Totally, 1807 reaction classes were identified. We manually inspected roughly 20 examples from each reaction type in the top 10 that were balanced by ChemBalancer (excluding nonclassified reactions) and could not find false positives. This means that all types of reactions were learned by the ChemMLM.

Solutions to (1) and (2) are therefore relegated to future work. We stress that the goal of this work was to propose an initial methodology and workflow that could help in balancing as a proof-of-concept and highlight limitations that can be addressed moving forward.

### The ChemMLM Completion Results

ChemMLM learned from the limited complete reactions and broadened the boundary of the complete reaction space to some originally incomplete reactions, still retaining the errors and noise present in the available complete reactions.

#### Model Training Results

**Figure 8:**
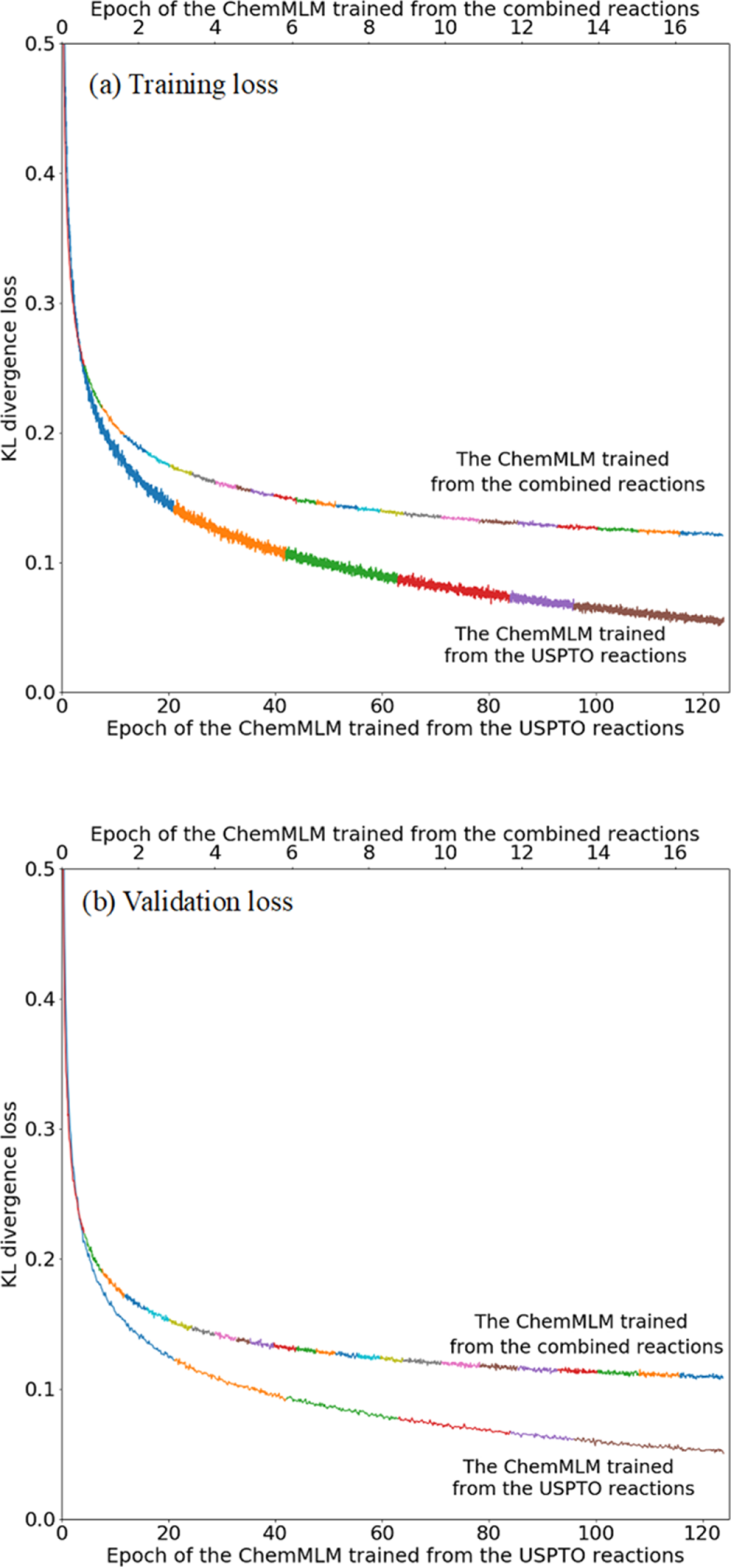
Training and validation KL divergence losses of the ChemMLM model learned from (a) USPTO reaction data set only and (b) combined reaction data set. The figure only shows the most optimal model using the training argument set stated in Table S2.

The ChemMLM models were trained from two data sources: the USPTO reactions and the combined reactions with Reaxys. Several attempts to tune training arguments were conducted for the ChemMLM learned from USPTO itself, while only one set of training arguments was conducted on the larger combined reaction data set, with the model configuration and the most optimal training arguments stated in the ChemMLM Model Training and Prediction section. As shown in Figure 8, using a large transformer model with approximately 83 million parameters to learn the large combined reaction data set with 7 million reactions, the model took approximately 25 days to complete 17 epochs of training, with each color in the training and validation loss curves corresponding to a single training day. The training and validation losses approach 0.121 and 0.118 eventually, respectively. As shown in the trend of the training and validation loss curves, the losses have not converged yet, and the learning process could continue. However, it was interrupted since this long training process could not reach as good performance as the ChemMLM model trained from the smaller USPTO data set in 6 days, which stopped at 0.050 and 0.052 training and validation losses, respectively, after 125 epochs. This was stopped because the validation loss started to be higher than the training loss. Potentially, the combined data set contains a higher level of semantic information, which could possibly train a better ChemMLM model. However, the ChemMLM model learned from the larger combined data set was not fine-tuned since this was computationally too expensive. The larger learning rate and batch size, as shown in Table S2 in the SI, were chosen for the model trained from the combined data set for the purpose of faster training.

#### Model Test Results

The models were assessed from the test data set of the two sources to determine the ratios of the correct prediction of the exact hidden molecules, at both sides of the reaction, as shown in Table 2.

**Table 2:** Ratios of Correct Prediction of the Exact Hidden Molecules with Short, Middle, and Long Token Lengths at the LHS and the RHS of the Reactions in the Test Reaction Data Set for the ChemMLM Models Trained from Two Complete Reaction Data Sources, USPTO and Combined Reaction Data Sets

models	molecule length	USPTO (%)	combined (%)
LHS ChemMLM	short	100	100
	middle	75.2	65.7
	long	24.5	1.8
RHS ChemMLM	short	99.8	100
	middle	81.3	62.4
	long	8.2	4.9

From the test data set, the model’s ability to predict short token length (token length = 1), middle token length (1 < token length ≤ 10), and long token length (token length > 10) hidden molecules was assessed, and the comparisons between exemplified predicted and ground truth hidden molecules at different lengths are shown in Table 3. The ChemMLM models could predict almost 100% of the ground truth hidden molecules at the short token length while retaining a very high correct rate at middle token lengths. However, the models were unable to predict long token length molecules, with predictions not only semantically incorrect but also invalid in SMILES syntax. For example, the last predicted molecule example shown in Table 3 has more right parentheses than left. This is because with longer token lengths in the hidden molecules, it becomes harder for the ChemMLM model to pick up the neighbor semantic context of the reaction SMILES strings since the very same neighbor tokens are also within the hidden molecule and uncertain.

**Table 3:** Comparison between the Exemplified Ground Truth Hidden Molecules and the Molecules at Different Token Lengths Predicted by the ChemMLM Models Trained from USPTO Complete Reactions

molecule length	prediction	ground truth
short	Cl	Cl
	O	O
	CO	CO
	CCCCO	CCCCO
middle	[H][H]	[H][H]
	CC(=O)O	CC(=O)O
	NC(=0)c1ncc(Br)cc1N	NC(=0)c1ncc(Br)cc1N
	O=C(CCl)Nc1	O=C(CCl)Nc1ccnnc1
long	CC=C(O)C1C)C(C(C)c1ccccc1	CC1=C(c2ccccc2)OC(C)(C)C1=O
	0 = C1CC1c2)2)c)c2c3ccccc3c2ccccc1	O=c1cc(-c2ccccc2)c2ccc3ccccc3c2[nH]1
	CCCCCCc1cn(CCS(=O)(=O)cc(CCccccc2)nn1	CCCCCCc1cn(CCS(=O)(=O)c2ccc(C)cc2)nn1
	CNC(=O)/C=C/[C1Cc1HCCCC CCCC)))))))))))(=))OCc1ccccc1	CNC(=O)/C=C/[C@H](Cc1ccccc1)NC(=O)[C@H](CCCNC(=O)OCc1ccccc1)NC(C)C

The longer token length molecules are longer than “a missing word” under the definition of the MLM. It fits better with another application of the BERT transformer model, the next-sentence prediction,^27,28^ which predicts the entire next sentence based on the previous context. This could be an area of exploration for the future prediction of longer molecules. However, this has not been implemented in this paper since the low model predictability of longer molecules would not significantly affect the ChemMLM’s ability to complete reactions. The missing molecules in an incomplete reaction are commonly the side reactants and side products, which are usually smaller molecules.

Table 2 also shows that the ChemMLM models do not show a significant difference in the prediction of hidden molecules between the LHS and RHS in terms of their correction rate of short, middle, and long token length molecules. This is because the models were trained using a dynamic mask method, which learned the semantics at two sides of the reactions simultaneously. However, the ChemMLM model trained from USPTO data shows better model performance in test data compared with the current ChemMLM model learned from the combined data. Perhaps, a fine-tuned ChemMLM model learned from the larger reaction data set could increase the correct prediction rate in the test data set, but the ChemMLM learned from the USPTO reactions was sufficiently predictive to predict the short and middle length hidden molecules. This indicates that the USPTO reaction data set is capable of providing comprehensive types of complete reactions for the ChemMLM model to learn from. The ChemMLM model in the rest of this paper is referred to as the ChemMLM trained from the USPTO reaction only.

#### Reaction Completion Results

**Figure 9:**
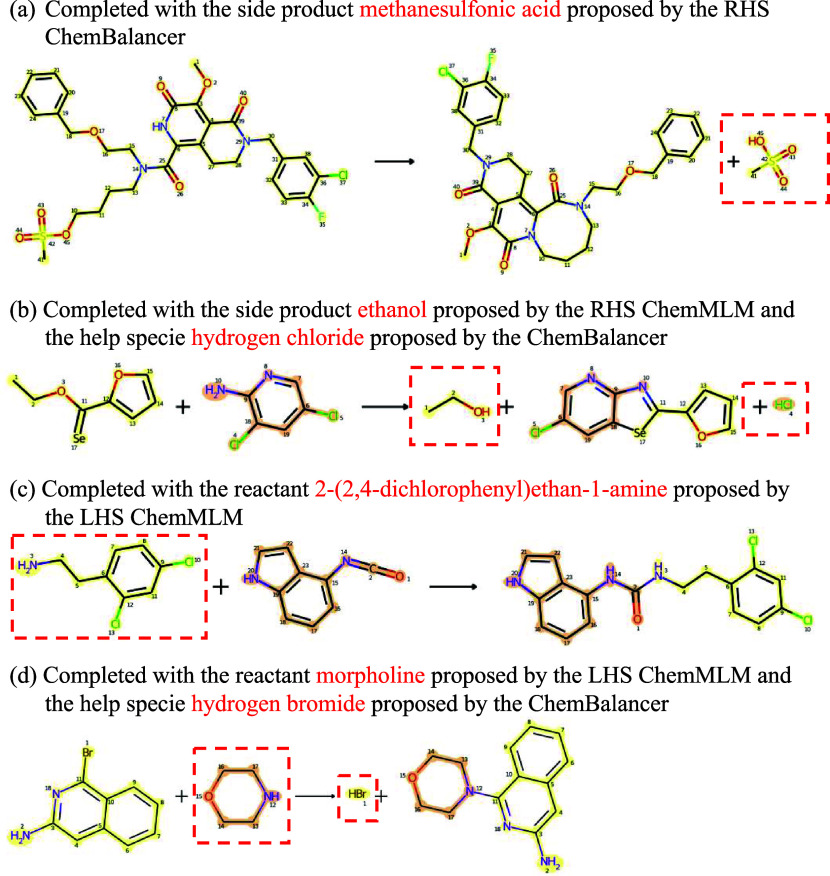
Examples of ChemMLM reaction completion results: (a) an RHS insufficient reaction completed by the RHS ChemMLM only, (b) an RHS insufficient reaction completed by the RHS ChemMLM plus ChemBalancer, (c) an LHS insufficient reaction completed by the LHS ChemMLM only, and (d) an LHS insufficient reaction completed by the LHS ChemMLM plus ChemBalancer. All reactions are atom-mapped by the RXNMapper and labeled with atom mapping indices.

For the incomplete reactions, ChemMLM proposes missing molecules at either the LHS or RHS based on ChemBalancer suggestions of LHS or RHS species insufficiency, and the proposed solutions are further checked by ChemBalancer. The exemplified reactions completed in this workflow are shown in Figure 9. In these examples, panels (a, c) show examples of reaction completion with the molecules proposed by ChemMLM itself, while panels (b, d) show examples of reaction completion by the molecules proposed by ChemMLM. All of the exemplified reactions were manually evaluated to be correct completions based on reaction mechanisms.

However, the reaction completion by ChemMLM still retains false positive reactions, with exemplified reactions shown in Figure 7b,c. In Figure 7b, the reactant is supposed to hydrolyze into two fragments, while the RHS ChemMLM predicts a chemically meaningless side product cyclohexyl 2-methylprop-2-enoate, and this updated reaction could be materially balanced with stoichiometric coefficients by ChemBalancer. Similarly, in Figure 7c, an incorrect side product ammonia was proposed by the RHS ChemMLM. With the help of species benzene proposed by ChemBalancer, the updated reaction could be materially balanced with stoichiometric coefficients. These false positive complete reactions were caused only in very rare situations. This happens when some of the 333 enumerations to predict the missing molecules by ChemMLM were not semantically meaningful but their SMILES syntax was valid. This prediction remains valid only when the updated reactions were possible to be balanced by large values of stoichiometric coefficients in coincidence.

For reaction completion, all incomplete reactions in the USPTO data set from the ChemBalancer subunit were passed to ChemMLM for the missing molecule prediction, while only 1% from Reaxys was randomly sampled and fed in ChemMLM. This is because the missing molecule prediction by ChemMLM was computationally expensive, which makes it harder to process the larger-scale Reaxys reaction data. Statistics for the reaction completion results are shown in Table 4, and this was concluded from all USPTO reactions and sampled Reaxys reactions. From Table 4, it is seen that a higher percentage of incomplete reactions could be completed from the USPTO than from the Reaxys reactions. This is very likely because a higher percentage of reactions in Reaxys were completed by ChemBalancer, as shown in Table 1, leaving higher difficulty for ChemMLM to predict missing molecules. Also, in general, the LHS species insufficient reactions were harder to complete by this workflow compared with the RHS species insufficient reactions.

**Table 4:** Statistics for the LHS and RHS Species Insufficient Reaction Completion Results by ChemMLM for the Reaction Records in USPTO and Reaxys Databases

reaction database	USPTO (%)	Reaxys (%)
completed reactions from LHS insufficient reactions	24.1	19.7
–completed by ChemMLM itself	8.2	6.7
–completed by ChemMLM and ChemBalancer reagents	0.4	4.9
–completed by ChemMLM and ChemBalancer help species	15.5	8.1
completed reactions from RHS insufficient reactions	42.7	33.4
–completed by ChemMLM itself	3.7	11.9
–completed by ChemMLM and ChemBalancer reagents	0.5	3.2
–completed by ChemMLM and ChemBalancer help species	38.5	18.3

Summarized from Tables 1 and 4, 3.3 and 7.2% of the total reactions from USPTO and Reaxys are originally complete/balanced reactions, respectively. 17.6 and 44.1% of total reactions in USPTO and Reaxys were completed, respectively, by the first method, ChemBalancer. Afterward, 34.8 and 16.8% of reactions were completed with the missing molecule prediction by the second method, ChemMLM. In total, 52.4% of USPTO and 60.9% of Reaxys were completed by the entire proposed method, from which the Reaxys percentage is an estimated number since only 1% of LHS and RHS species insufficient reactions were sampled and fed to the ChemMLM model. Moreover, a small portion of the completed reactions is false positive completions, and those reactions could not be differentiated currently unless they were manually removed from the completed reactions. Since we kept a ChemBalancer at the downstream of the ChemMLM to detect if the suggested molecule by the ChemMLM could potentially balance the reaction, these results given from this workflow would be either correct answers or false positives given by the downstream ChemBalancer. The other invalid results are all removed from the various safeguard steps of the workflow. This makes the qualitative analysis of the ChemMLM results similar to the results of the upstream ChemBalancer, Therefore, the future improvement of the ChemBalancer would eventually be beneficial to filter the incorrect ChemMLM results.

## Conclusions

Current reaction data records from multiple reaction databases, including USPTO and Reaxys, are missing important reaction participating species and stoichiometric coefficients. Completing reaction structures in the reaction data would make it possible to understand the true molecular flow for the reaction routes in CASP tasks. Although multiple tools such as rule-based reaction templates, atom mapping software, etc., have been developed to investigate molecular transformations in reactions, identifying the missing molecules in reactions remains a challenging task, since most of these data-driven tools were developed from the incomplete reaction data sets. The reaction completion challenge could not be fully solved unless a “golden data set” with comprehensive complete reactions appears. With the limited complete reaction data sources, the objective of this work was to learn from these limited complete reactions and broaden the boundary of the complete reaction space to some originally incomplete reactions toward the reaction structure completion of the current reaction records.

To do this, a workflow including both heuristics and machine learning methods was proposed. From USPTO and Reaxys reaction data, a heuristic-based balancing algorithm, namely, ChemBalancer, was developed to investigate if a reaction is balanced based on atom equality of each element and electron balance at the two sides of the reaction. ChemBalancer also attempted to complete the imbalanced reactions by adding molecules from reagents, solvents, and a list of the most frequently appearing small molecules in reactions. The remaining incomplete reactions were classified as either LHS or RHS species insufficient reactions. ChemBalancer identified 3.3 and 7.2% originally complete/balanced reactions from USPTO and Reaxys, respectively, and further completed 14.3 and 36.9% of the total reactions by adding possible missing molecules to the reactions.

A machine learning BERT transformer model, namely, ChemMLM, was developed to learn from the semantic meaning of the reaction SMILES strings from the complete reaction data set identified from the last step. Using the masked language model scheme, analogous to missing words in sentences, ChemMLM was trained to predict the possible missing molecules for the incomplete reactions. From the test data set, it is proven that ChemMLM was confident to predict short and middle (token length ≤ 10) length missing molecules. Given the classification of LHS or RHS species insufficient of the incomplete reactions, ChemMLM either predicted missing molecules to the LHS or RHS. ChemMLM further completed 34.8 and 16.8% reactions from the total USPTO and Reaxys reaction data set. The entire workflow completed 52.4 and 60.9% of reactions from USPTO and Reaxys in total, retaining with false positive complete reactions that could be only manually detected.

For future works, to improve the prediction accuracy of longer-length molecules, as a longer-length molecule (token length > 10) can have molecular SMILES strings analogous to a sentence, the next-sentence prediction scheme of the BERT transformer could potentially be used to predict these molecules. Similarly, the development of GPT^29^ in the language model may benefit in reaction completion. As the decoder part of the transformer, the “attention” scheme of the GPT expertise in longer-length text generation.^29^ However, since most incomplete reactions are missing side reactants and products, which are smaller molecules, this would not significantly increase the reaction completion rate. Moreover, to minimize the false positive rate in the completed reactions, a calibration step could be added. Atom mapping confidence score would be an important index to understand the atom rearrangement in reactions. However, since there is no ground truth atom mapping method, this index could only be used as a recommendation for false positive detection. A more promising atom mapping tool is required to calibrate the model-completed reactions. Eventually, to ultimately achieve reaction structure completion, a “golden data set” is called for. This requires a more accurate NLP text-mining tool to reduce the noise when mining reactions from the literature and manual curation of at least a few reactions from each reaction subcategory.

## Data Availability

USPTO and Reaxys databases were used in this study. USPTO data are available via https://figshare.com/articles/dataset/Chemical_reactions_from_US_patents_1976-Sep2016_/5104873. Reaxys data were made available to this study via Elsevier R&D Collaboration Network and are available to readers from Elsevier via a license. The code used for the ChemBalancer and the ChemMLM can be found online at https://github.com/chonghuanzhang/balancing_rxn.

## Supporting Information Available

Help species in the ChemBalancer and training arguments for the ChemMLM models and reaction classes statistics in the USPTO reaction data set (PDF)

## References

[ref1] Elsevier Reaxys. https://www.reaxys.com/. (accessed February 06).

[ref2] Lowe D. Chemical Reactions from US Patents, 2023. https://figshare.com/articles/dataset/Chemical_reactions_from_US_patents_1976-Sep2016_/5104873. (accessed May 02, 2023).

[ref3] Weber J. M.; Guo Z.; Zhang C.; Schweidtmann A. M.; Lapkin A. A. Chemical data intelligence for sustainable chemistry. Chem. Soc. Rev. 2021, 50 (21), 12013–12036. 10.1039/D1CS00477H. 34520507

[ref4] Kearnes S. M.; Maser M. R.; Wleklinski M.; Kast A.; Doyle A. G.; Dreher S. D.; Hawkins J. M.; Jensen K. F.; Coley C. W. The Open Reaction Database. J. Am. Chem. Soc. 2021, 143 (45), 18820–18826. 10.1021/jacs.1c09820. 34727496

[ref5] Szymkuć S.; Gajewska E. P.; Klucznik T.; Molga K.; Dittwald P.; Startek M.; Bajczyk M.; Grzybowski B. A. Computer-Assisted Synthetic Planning: The End of the Beginning. Angew. Chem., Int. Ed. 2016, 55 (20), 5904–5937. 10.1002/anie.201506101. 27062365

[ref6] Synthia Synthia Organic Retrosynthesis Software. https://www.sigmaaldrich.com/GB/en/technical-documents/technical-article/chemistry-and-synthesis/organic-reaction-toolbox/resources#ref2. (accessed February 09).

[ref7] Coley C. W.; Barzilay R.; Jaakkola T. S.; Green W. H.; Jensen K. F. Prediction of Organic Reaction Outcomes Using Machine Learning. ACS Cent. Sci. 2017, 3 (5), 434–443. 10.1021/acscentsci.7b00064. 28573205 PMC5445544

[ref8] Jaworski W.; Szymkuć S.; Mikulak-Klucznik B.; Piecuch K.; Klucznik T.; Kaźmierowski M.; Rydzewski J.; Gambin A.; Grzybowski B. A. Automatic mapping of atoms across both simple and complex chemical reactions. Nat. Commun. 2019, 10 (1), 1434 10.1038/s41467-019-09440-2. 30926819 PMC6441094

[ref9] Schwaller P.; Hoover B.; Reymond J.-L.; Strobelt H.; Laino T. Extraction of organic chemistry grammar from unsupervised learning of chemical reactions. Sci. Adv. 2021, 7 (15), eabe4166 10.1126/sciadv.abe4166. 33827815 PMC8026122

[ref10] Nugmanov R. I.; Mukhametgaleev R. N.; Akhmetshin T.; Gimadiev T. R.; Afonina V. A.; Madzhidov T. I.; Varnek A. CGRtools: Python Library for Molecule, Reaction, and Condensed Graph of Reaction Processing. J. Chem. Inf. Model. 2019, 59 (6), 2516–2521. 10.1021/acs.jcim.9b00102. 31063394

[ref11] Patel H.; Bodkin M. J.; Chen B.; Gillet V. J. Knowledge-based approach to de novo design using reaction vectors. J. Chem. Inf. Model. 2009, 49 (5), 1163–1184. 10.1021/ci800413m. 19382767

[ref12] Weininger D. SMILES, a chemical language and information system. 1. Introduction to methodology and encoding rules. J. Chem. Inf. Comput. Sci. 1988, 28 (1), 31–36. 10.1021/ci00057a005.

[ref13] Devlin J.; Chang M.-W.; Lee K.; Toutanova K. In BERT: Pre-Training of Deep Bidirectional Transformers for Language Understanding, Conference of the North American Chapter of the Association for Computational Linguistics: Human Language Technologies; Association for Computational Linguistics: Minneapolis, MN, 2019; pp 4171–4186.

[ref14] Arun A.; Guo Z.; Sung S.; Lapkin A. A. Reaction Impurity Prediction using a Data Mining Approach**. Chem.–Methods 2023, 3, e202200062 10.1002/cmtd.202200062.

[ref15] Dahlgren B. ChemPy: A package useful for chemistry written in Python. J. Open Source Software 2018, 3 (24), 565 10.21105/joss.00565.

[ref16] Salazar J.; Liang D.; Nguyen T. Q.; Kirchhoff K. Masked Language Model Scoring. arXiv:1910.14659, arXiv.org e-Print archive, 2019. https://arxiv.org/abs/1910.14659.

[ref17] Liu Y.; Ott M.; Goyal N.; Du J.; Joshi M.; Chen D.; Levy O.; Lewis M.; Zettlemoyer L.; Stoyanov V. Roberta: A Robustly Optimized BERT Pretraining Approach. arXiv:1907.11692, arXiv.org e-Print archive, 2019. https://arxiv.org/abs/1907.11692.

[ref18] Schwaller P.; Laino T.; Gaudin T.; Bolgar P.; Hunter C. A.; Bekas C.; Lee A. A. Molecular Transformer: A Model for Uncertainty-Calibrated Chemical Reaction Prediction. ACS Cent. Sci. 2019, 5 (9), 1572–1583. 10.1021/acscentsci.9b00576. 31572784 PMC6764164

[ref19] Gage P. A new algorithm for data compression. C Users J. 1994, 12 (2), 23–38.

[ref20] Sennrich R.; Haddow B.; Birch A. In Neural Machine Translation of Rare Words with Subword Units, Proceedings of the 54th Annual Meeting of the Association for Computational Linguistics; Association for Computational Linguistics: Berlin, Germany, 2016; pp 1715–1725.

[ref21] Thakkar A.; Kogej T.; Reymond J.-L.; Engkvist O.; Bjerrum E. J. Datasets and their influence on the development of computer assisted synthesis planning tools in the pharmaceutical domain. Chem. Sci. 2020, 11 (1), 154–168. 10.1039/C9SC04944D. 32110367 PMC7012039

[ref22] Face H. AI Community Building the Future. 2022.

[ref23] Google Google Colab. 2022.

[ref24] Hendrycks D.; Gimpel K. Gaussian Error Linear Units (Gelus). arXiv:1606.08415, arXiv.org e-Print archive, 2016. https://arxiv.org/abs/1606.08415.

[ref25] RDKit: Open-Source Cheminformatics. http://www.rdkit.org. (accessed February 06).

[ref26] Jiang X.; Röcker C.; Hafner M.; Brandholt S.; Dörlich R. M.; Nienhaus G. U. Endo-and exocytosis of zwitterionic quantum dot nanoparticles by live HeLa cells. ACS Nano 2010, 4 (11), 6787–6797. 10.1021/nn101277w. 21028844

[ref27] Sun Y.; Zheng Y.; Hao C.; Qiu H. NSP-BERT: A Prompt-based Zero-Shot Learner Through an Original Pre-training Task--Next Sentence Prediction. arXiv:2109.03564, arXiv.org e-Print archive, 2021. https://arxiv.org/abs/2109.03564.

[ref28] Shi W.; Demberg V. Next Sentence Prediction Helps Implicit Discourse Relation Classification Within and Across Domains, Proceedings of the 2019 Conference on Empirical Methods in Natural Language Processing and The 9th International Joint Conference on Natural Language Processing (EMNLP-IJCNLP); Association for Computational Linguistics, 2019; pp 5790–5796.

[ref29] Brown T. B.; Mann B.; Ryder N.; Subbiah M.; Kaplan J.; Dhariwal P.; Neelakantan A.; Shyam P.; Sastry G.; Askell A.; Agarwal S.; Herbert-Voss A.; Krueger G.; Henighan T.; Child R.; Ramesh A.; Ziegler D. M.; Wu J.; Winter C.; Hesse C.; Chen M.; Sigler E.; Litwin M.; Gray S.; Chess B.; Clark J.; Berner C.; McCandlish S.; Radford A.; Sutskever I.; Amodei D. Language Models are Few-Shot Learners. arXiv:2005.14165, arXiv.org e-Print archive, 2020. https://arxiv.org/abs/2005.14165.

