# Supplementary material for: Completing and Balancing Database Excerpted Chemical Reactions with a Hybrid Mechanistic-Machine Learning Approach
Source: ACS Omega. 2024 Apr 10;9(16):18385–99. doi: 10.1021/acsomega.4c00262 (PMC11044172; doi:10.1021/acsomega.4c00262)
Supplement: Supplementary file 1 — ao4c00262_si_001.pdf [file ao4c00262_si_001.pdf]

Completing and balancing database excerpted chemical reactions with a hybrid mechanistic - machine learning approach

Electronic Supplementary Information

*Chonghuan Zhang,[1] Adarsh Arun[1,2,3] and Alexei A. Lapkin[1,2,3] **

[1] Department of Chemical Engineering and Biotechnology, University of Cambridge, Philippa Fawcett Drive, Cambridge CB3 0AS, United Kingdom

[2] Cambridge Centre for Advanced Research and Education in Singapore, CARES Ltd, 1 CREATE Way, CREATE Tower #05-05, 138602 Singapore

[3] Chemical Data Intelligence (CDI) Pte Ltd, 80 Robinson Road, #02-00, 068898, Singapore

∗ Corresponding author E-mail addresses: aal35@cam.ac.uk

Table of Contents

S1 Help species in the ChemBalancer 3

S2 Training arguments for the ChemMLM models 3

S3 Reaction classes statistics in the USPTO reaction dataset 4

S1 Help species in the ChemBalancer

The help species are added sequentially to the RHS of a reaction and used to balance the reaction in the ChemBalancer. The reaction is defined as complete with help species if it is balanced with the addition of these species to the RHS. The help species are shown in Table S1.

Table S1. The library of help compounds.

**Categories**	**Molecules**
Ion	sodium ion, potassium ion, nitronium ion, sulphate ion
Oxyacid	phosphoric acid, sulfuric acid, nitric acid, chloric acid
Alcohol	methanol, ethanol, propanol, butanol
Carboxylic acid	acetic acid, propanoic acid, butanoic acid, methoxyacetic acid
Aromatic compound	methane, benzene, toluene, phenol, chlorobenzene
Others	water, hydrogen, oxygen, ammonia, nitrogen
Hydrogen acid	hydrogen chloride, hydrogen bromide, hydrogen iodide, hydrogen fluoride, phosphine, hydrogen sulfide

S2 Training arguments for the ChemMLM models

The training arguments for the two ChemMLM models, learned from USPTO data and combined data are shown in Table S2.

Table S2. The training arguments for two ChemMLM models, learned from USPTO data and combined data respectively.

**ChemMLM**	**USPTO**	**Combined**
Epoch	175	17
Learning rate	10^{-4}	1.5×10^{-4}

Gradient accumulation steps	1	1
Batch size	16	32

S3 Reaction classes statistics in the USPTO reaction dataset

Figure S1 below shows a chart with the top 10 most common reaction classes in the USPTO dataset we used, based on the NameRXN tool developed by NextMove software. Totally, 1807 reaction classes were identified. Note that 21 % of the dataset could not be categorized (reaction class of 0.0) and this is provided in addition to the top 10 classes.

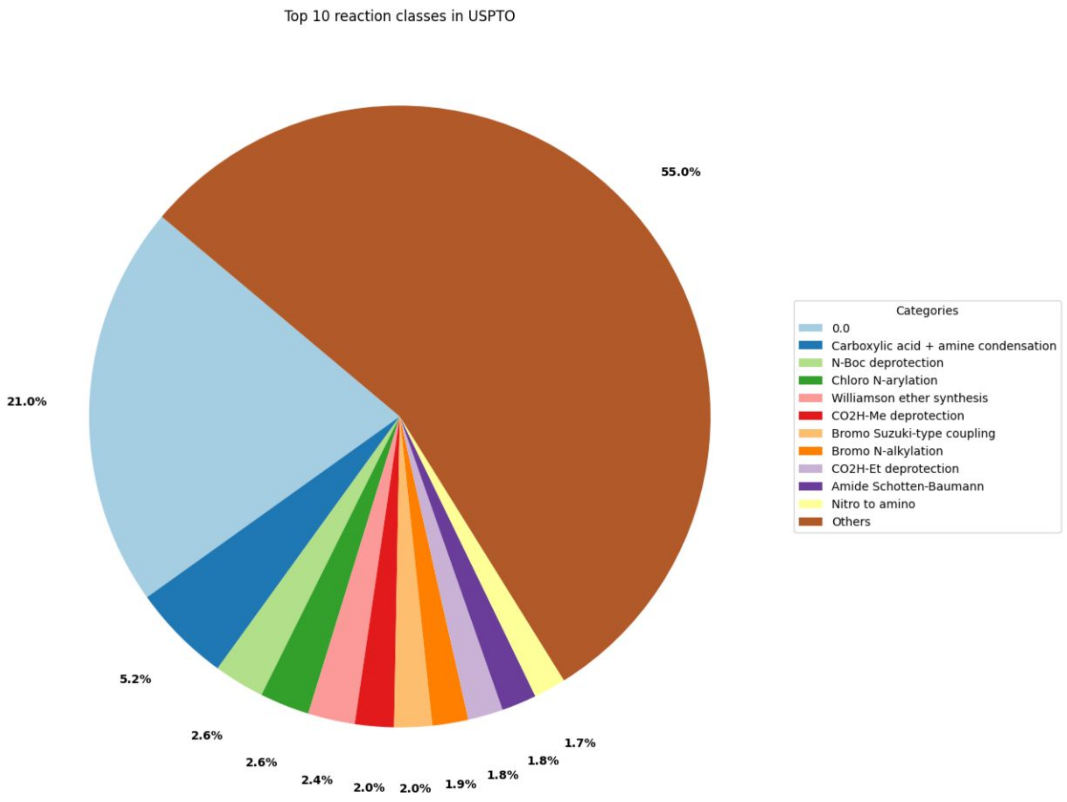

Figure S1 A chart showing the top 10 reaction classes in the USPTO dataset used in the manuscript based on the NameRXN tool developed by NextMove software. 21 % of the dataset could not be classified (0.0); this is provided in addition to the top 10. Carboxylic acid and amine condensation is the most prevalent reaction type. 55 % of the dataset is categorized as others and contains ~1796 reaction classes (<1.7 % per class).

Contents of the materials supplied as Supporting Information

1. Help species in the ChemBalancer
2. Training arguments for the ChemMLM models
3. Reaction classes statistics in the USPTO reaction dataset
